# Chemical composition and pharmacological significance of *Anethum Sowa* L. Root

**DOI:** 10.1186/s12906-017-1601-y

**Published:** 2017-02-23

**Authors:** Md Moshfekus Saleh-e-In, Nasim Sultana, Md Matiur Rahim, Md Aminul Ahsan, Md Nurul Huda Bhuiyan, Md Nur Hossain, Md Mahbubar Rahman, Sudhangshu Kumar Roy, Md Rabiul Islam

**Affiliations:** 1 Department of Chemistry, Jahangirnagar University, Savar, 1342 Dhaka, Bangladesh; 2 Institute of National Analytical Research and Services, BCSIR Laboratories, Bangladesh Council of Scientific and Industrial Research, Dhaka, 1205 Bangladesh; 3 Food Toxicology Research Section, Institute of Food Science and Technology, Bangladesh Council of Scientific and Industrial Research, Dhaka, 1205 Bangladesh; 4 Industrial Microbiology Research Division, Institute of Food Science and Technology, Bangladesh Council of Scientific and Industrial Research, Dhaka, 1205 Bangladesh; 5 Plant Protein Research Division, IFST, Bangladesh Council of Scientific and Industrial Research, Dhaka, 1205 Bangladesh; 6 IFRD, Bangladesh Council of Scientific and Industrial Research, Dhaka, 1205 Bangladesh

**Keywords:** *Anethum sowa* L, Amino acid, Apiol, Biological activity, Fatty acid, ICP/MS, Thermal analysis

## Abstract

**Background:**

Medicinal herbs are used for the treatment of different ailments since antiquity. Different parts of *Anethum sowa* L. is used in folk medicine as a carminative for the treatment of flatulence, colic and hiccups of infants and children, antioxidant, antimicrobial and antispasmodic agent. The aim of our present study is to evaluate the chemical composition of the essential oil, proximate and elemental composition, amino acid, fatty acid profile and thermal behaviour of its root part as well as different pharmacological activities like antioxidant, antimicrobial and cytotoxicity of the root essential oil.

**Methods:**

The air-dried roots of *Anethum sowa* L. were subjected to hydro-distillation to yield the essential oil. The antioxidant activity of the essential oil was studied by DPPH radical scavenging activity. The antimicrobial activity was tested against four Gram-positive, six Gram-negative bacteria and four fungi species. The minimum inhibitory concentration (MIC) and Minimum bacterial concentration (MBC) for each examined microorganism were determined using the micro-dilution method. The LC_50_ value of the oil was also evaluated by brine shrimp lethality assay. The subsequent proximate analysis was also done by AOAC methods. The elemental analysis of the root powder was analysed by ICP-MS, AAS and FP system. The fatty acid was extracted by hot and cold extraction method and the analyses were carried out by GC. The amino acid profile was done by the amino acid analyzer. The DTA, DTG and TG of the root powder were taken by the thermogravimetric analyzer.

**Results:**

A total of 24 constituents was identified and quantified in the essential oil and its water extract portion by GC and GC-MS. Apiol (81.99 and 74.779%) was found the highest phenylpropanoid constituent followed by m-diaminobenzene (10.446 and 8.778%) in the essential oil and aqueous extract portion. On the other hand, β-butyrolactone (5.13%) and isobutyl acetone (3.73%) were found in the major constituents in the water extract part. The IC_50_ value of the essential oil was found to be 3.07 mg/mL by DPPH radical assay methods. The LC_50_ value of the brine shrimp cytotoxicity assay of the essential oil was observed at 0.81 μg/mL. The essential oil showed better activity on Gram-negative bacteria than Gram-positive bacteria and fungi. The proximate composition showed that root contained 5.29% ash, 2.01% protein, 54.09% crude fibre, 0.15% essential oil and 1.14% fatty oil for hot extract and 0.23% for cold extract on the dried basis. The palmitic (33.81 & 31.58%) and linoleic acid (30.03 & 23.79%) were the major saturated and unsaturated fatty acids in the cold and hot extracted root powder respectively. Ca (23,600 mg/kg), Mg (7620.33 mg/kg) and K (1286.15 mg/kg) were the most predominant elements followed by Ni (1187.30 mg/kg), Se (913.79 mg/kg), Li (317.84 mg/kg), Na (288.72 mg/kg) and Fe (206.88 mg/kg). The toxic elements were found to be within the permissible limit. Glutamic acid (19.37%), glycine (14.53%) and lysine (17.08%) were found as the major amino acids. The decomposition rates were obtained by TG, DTG and DTA curve of the powder sample at various temperature ranges.

**Conclusions:**

The results demonstrated that the root part of *Anethum sowa* L. is a rich source of mineral elements, essential amino acid and fatty acids. The essential oil is the highly potential as bioactive oil for pharmaceuticals and medical applications, possessing antioxidant, antimicrobial and cytotoxic activities. The thermal analysis suggested as a simple, effective and rapid method to characterize the *Anethum sowa* L. species as well as to assess for herbal formulation.

**Electronic supplementary material:**

The online version of this article (doi:10.1186/s12906-017-1601-y) contains supplementary material, which is available to authorized users.

## Background

Herbs and spices, grown wildly in various regions of the world have been used for culinary and medicinal purposes since ancient times. *Anethum sowa* L. (Bengali-Shulfa) belonging to the family *Apiaceae* (*Umbelliferae*), comes under genus *Anethum* and it is an annual and winter spice crop in Bangladesh. It is mostly grown in the northern part of Bangladesh. A variant called Indian dill or sowa (*Anethum sowa* Roxb.) is largely cultivated in Bangladesh, India, Egypt and Japan. Indigenous people consume it as a spice for a flavouring agent in food preparation. The herb grows ordinarily 2–2.5 ft. in height with small feathery leaves, tapped and branched roots [1, 2]. The chemical composition of the essential oil of the two chemotypes i.e. European dill (*Anethum graveolens* L.) and Indian dill (*Anethum sowa* L.) are differentiated mainly by the apiol and carvone content. *Anethum sowa* is rich in apiol whereas *Anethum graveolens* is rich in carvone. The typical flavor of dill herb oil is due to α-phellandrene, limonene and dill ether (anethofuran) [3]. The green herb, seeds and its roots are used as folkloric medicine e.g. aromatic, carminative especially useful in the treatment of flatulence, colic and hiccups of infants and children [4]. Recently, it has been reported that seed essential oils are the potential source of antioxidant and also have antimicrobial and antispasmodic properties [5].

Due to commercial interest, extensive studies have been done on dill seed (fruit) and weed (herb) essential oil for its sensory qualities, physical and chemical composition. Very few reports [6–9] have been found in the essential oil and mineral content of the root part of *Anethum graveolens* L (Dill) species to the best of our knowledge. But the chemical composition and pharmacological studies of *Anethum sowa* root are still to the unveiled. Therefore, the aims of this study were to elucidate the chemical composition as well as its pharmacological activities of the essential oil, elemental composition, amino acid, fatty acid profile and thermal behavior of its root part. This information can be helpful in the formulation of various herbal forms of medicines.

## Methods

### Plant material

Whole plants were collected from Keranigonj, Dhaka of Bangladesh after harvesting. The plants were taxonomically classified and identified scientifically by Dr. Sardar Nasir Uddin, PSO, Bangladesh National Herbarium Centre, Dhaka, Bangladesh. A voucher specimen of the plant was preserved in Bangladesh National Herbarium Centre with the Accession Number-31,282. Root samples were chopped into small pieces, dried in a shade by air and ground to powder by grinding machine (100 mesh) and finely stored in an air tight high-density polyethylene bag.

### Physico-chemical and proximate studies

The physicochemical and proximate studies were carried out with three replications by the standard methods [10–12].

### Extraction of fatty acids

Fatty acid extraction of the root powder was carried out by Soxhlet apparatus with hexane in a temperature controlled heating mantle for 72 h (working time) as per Soxhlet method [13] and simultaneously a cold extraction was carried out successively by petroleum ether (b.p. 40–60 °C) at room temperature. All extractions were carried out three times and evaporated the residual solvent by Buchi rotary evaporator at low temperature and reduced pressure.

### Fatty acid analysis

The fatty acid composition was determined by analysis of their methyl esters. The fatty acid methyl esters (FAMEs) of the hot and cold extracted fatty acids were prepared through esterification by using BF_3_. MeOH complex, according to the AOAC method [14].

### Instrument and separation conditions of the fatty acids

Fatty acid methyl esters were analysed and identified by using Shimadzu 2025 Gas Chromatograph with a Flame Ionization Detector and GC Work Station Software. A fused capillary column AT™-1 (30 m × 0.25 mm I.d. and 0.1 μm film thickness) was used for separation. Helium was used as carrier gas, at a flow rate of 2 mL/min. Injection and detector temperature was 250 °C. The oven temperature was programmed at 120 °C −270 °C by raising the temperature at 7 °C/min. Split ratio was 80. The fatty acid compounds were identified by comparison of relative retention times and peak positions of the standard fatty acid chromatogram. Atherogenic Index (AI) and Thrombogenic Index (TI) were calculated according to Ulbricht and Southgate [15] which AI is C_12:0_ + (4 × C_14:0_) + C_16:0_/PUFA + MUFA and TI is C_14:0_ + C_16:0_ + C_18:0_/0.5 × MUFA + 0.5 × PUFA.

### Amino acid analysis

The root powder sample (5.0 g) was refluxed with 6 M HCl (50 mL) at 110 °C for 24 h for protein hydrolysis. Then the solution was kept in an evaporating dish to evaporate HCl on a water bath. The residue was again dissolved in 20 mL 0.1 M HCl and evaporation was repeated twice to remove excess of the acid. It was then filtered through Whatman No. 1 filter paper with 0.1 M HCl in a volumetric flask which was known as a stock solution. Again, the stock solution was filtered through 0.45 μm syringe filter with pressure for amino acid analysis [16]. Then the stock and standard solutions were run through Shimadzu amino acid analyzer with a fluorescent detector. The sample amino acids were identified by comparing with standard amino acids retention times and calculated the percentage of their individual areas.

### Elemental analyses

#### Reagents, water and standard

Nitric acid (HNO_3_) of trace metal analysis grade, hydrochloric acid (HCl) 37% of BDH, analar grade and high purity de-ionized water from the Barnstead purification system were used throughout the study. Multi-element stock solutions containing 10 mg/L of each element were obtained from USA (Accu Trace TM Reference Standard).

*ICP*-*MS tuning solution*: Contains 10 ppb Ba, Be, Ce, Co, In, Pb, Mg, Tl and Th for instrument tuning and verification of performance.

*Metals stock standard for ICP*-*MS*:10 mg/L (Reference / Traceable) of metals Be, Bi, Cd, Cs, Cr, Co, Cu, Ga, In, Li, Ni, Pb, As, Se and Ag.

*Preparation of working standard for calibration*: Working standard were prepared of 1, 5, 10, 20 and 50 μg/L from 100 μg/L intermediate standard by 1% HNO_3_ diluents for carrying out analysis.

### Ashing procedure and sample preparation for ICP-MS and AAS

A certain amount of moisture less sample powder was taken. Ashing and subsequent sample preparation were performed as per AOAC method [10].

### ICP-MS instrument and operating condition

The elemental analyses were done by Varian UltraMass™ ICP-MS system (Varian Optical Spectroscopy Instruments, Melbourne Australia). The plasma source was 99.998% argon (Carbagas 3097, Liebefeld, Bern, Switzerland). All operating parameters were under computer control. Operating conditions of the instrument are depicted in Table 1.

**Table 1:** Operating conditions of Varian ICP-MS

Parameters	Settings
*Plasma conditions*	
RF power	1.40 kW
Plasma Ar flow rate.	18.0 L min^−1^
Auxiliary Ar flow rate	2.25 L min^−1^
Sheath gas flow	0.20 L min^−1^
Nebulizer gas flow	1.0 L min^−1^
Sampling depth	6.50 mm
Pump Rate	5 rpm
*Instrument*	
Sampler cone: Nikel	1.0 mm orifice diameter
Skimmer cone: Nikel	0.5 mm orifice diameter

### Atomic absorption spectrometry (AAS) analyses

Mg, Fe, Mn and Ca were analysed by Flame Atomic Absorption Spectrometer (Varian AA 240 FS). Al was analyzed by Zeeman Atomic Absorption Spectrometer (Varian AA 240 Z) in graphite furnace and the total Hg was analyzed by cold vapor hydrate generation Atomic Absorption Spectrometer (Varian AA 220 FS) followed by the Varian operating manual. The metal stock standard for Mg, Fe, Ca, Al and Mn were 1000 mg/L (Reference/Traceable) and quantification limit for the elements were at ppm levels. The detection limit for Fe, Mn and Al are 0.027, 0.005 and 0.00196 mg/mL respectively. On the other hand, Hg stock standard was AR grade of equivalent 100 mg/L. The quantification limit for the total Hg was 0.01 μg/L (ppb). All calibrated standard, quality control standard and check standard are traceable to National Institute of Standard and Technology (NIST). Recovery of quality spiked sample, duplicate samples and quality control sample were observed. The recovery ranges for each parameter were 100 ± 10%.

### Flame photometry analyses

The concentration of Na and K were analysed by Flame Photometer (Jenway PFP-7, England, UK), CRM (Certified Reference Material). Standard (ranging from 1 to 5 ppm) of Na^+^ and K^+^ solution were used in the serial dilution method for standard curve within linear calibration range and total quantities in samples were calculated.

### Thermogravimetric analysis

TG/DTA 6300 (The Seiko Instrument, USA) thermo gravimetric analyzer was used for monitoring continuous weight change of root powder due to drying, volatilisation and gasification and followed a linear heating program. The DTG and DTA curves were recorded in terms of percentage weight loss per minute and temperature difference in mV (for DT). Powder sample (7.34 mg) was heated in a platinum crucible under the furnace atmosphere at a heating rate of 20 °C/min up to the final temperature performed of 800 °C. The α-Al_2_O_3_ (6 mg) was used as a reference material.

### Extraction of essential oils

*A. sowa* root was subjected to hydro-distillation using Clevenger’s apparatus [17] for 4 h. Emulsified water soluble constituents were extracted with ethyl acetate and dried under high vacuum rotary evaporator at 35^0^ C. Both oils were then dried over anhydrous magnesium sulphate and stored at 4 °C prior to analysis.

### GC-MS analysis

The essential oils were analysed by Electron Impact Ionization (EI) method on GC-2010 Shimadzu Gas Chromatograph, coupled to a GC-MS QP 2010 plus Shimadzu Mass Spectrometer fitted with RTX-5 MS fused silica capillary column (Supelco Inc.) (30 m × 2.5 mm; 0.25 μm film thickness). The column temperature was 40 °C (hold 2 min) to 220 °C (hold 5 min) at the rate of 10 °C/min, maintained with carrier gas helium at a constant pressure of 90 kPa (Acquisition parameters full scan; scan range 40–550 amu). The split ratio was 10. Mass spectra were taken at 70 eV.

### Identification of the compounds

The constituents of the essential oil were identified by retention indices under temperature-programmed conditions based on co-injection of homologous n-alkanes (C_6_-C_24_) on the RTX-5 MS capillary column. Compounds were identified by comparison of their mass spectra with those of the internal reference mass spectral NIST-107 library.

### Antioxidant activity

The antioxidant activity of the root essential oil on the stable radical 1, 1-diphenyl-2-picrylhydrazyl (DPPH) (0.2 Mmol) (Sigma-Aldrich) was determined by the Brand-Williams method [18] with some modifications. The oil was dissolved in methanol and applied concentrations were 46.5 to 0.36328 mg/mL by serial dilution technique. Ascorbic acid (ASA) and tert-butyl-1-hydroxytoluene (BHT) were used as positive control. The absorbance was measured at 517 nm using an Analytic Jene Spekel 1300 UV spectrophotometer. The per cent of inhibitions were calculated from the equation:

$$ \%\ \mathrm{inhibition} = \left(1\hbox{-}\ {\mathrm{ABS}}_{\mathrm{sample}}/{\mathrm{ABS}}_{\mathrm{control}}\right)\times 100 $$

The data of IC_50_ values were transformed into a straight line by means of a trend line fit linear regression analysis by MS Excel version 7 Software for windows.

### Cytotoxic activity of brine shrimp lethality bioassay

In vitro Brine Shrimp lethality bioassay of the essential oil was exploited to detect cytotoxicity by Mayer’s method [19]. Brine Shrimp eggs were hatched in the brine solution (3.8% NaCl in distilled water) within 48 h. The samples were prepared by dissolving in DMSO solution (not more than 50 μL in 5 mL solution) and it was applied in 5 mL brine solution to attain the concentrations of 0.0195 to 10.0 μg/mL. DMSO (50 μL) was used as negative control. Standard vincristine sulphate (VcS) (0.078 μg/mL to 10.0 μg/mL) was used as positive control. Approximately 10 matured nauplii were taken to each vial. The numbers of surviving nauplii were counted after 24 h. The lethal concentrations (LC_50_) and the dose-response data were calculated by Biostate 2007 data analysis software.

### Antimicrobial activity test

The antimicrobial activity of the essential oil was determined by diffusion and dilution method [20] against 4 gram-positive (*Bacillus subtilis*, *Staphylococcus aureus*, *Bacillus cereus*, *Enterococcus faecalis*), 6 gram-negative bacteria (*Salmonella typhi*, *E. coli 12079*, *E. coli 2799*, *Pseudomonas aeruginosa*, *Salmonella enteritidis*, *Acetobacter aceti*) and 4 fungi (*Candida albicans*, *Aspergillus niger, Sacharomyces cerevaceae* and *Trichoderma sp.*) species.

### Diffusion method

The test microbes were taken from the broth culture with an inoculating loop and transferred to the test tubes containing 5.0 mL sterile distilled water. The microbial suspension turbidity was adjusted to McFarland standard number 0.5, in Mueller Hinton Broth (Himedia, India). A cotton swab was then used to inoculate the test tube suspension onto the surface of the Mueller Hinton agar plates and the plates were allowed to dry. The agar was allowed to set and harden. Holes were made by using a sterile cork borer from each petri-plate to ensure proper distribution of holes (cups) in the periphery and one in the centre. Agar plugs were removed. Different cork borers were used for different test organisms. Two standard discs (6 mm in diameter) were transferred onto the agar surface by using sterile forceps. Each hole was impregnated with 40 μL of a sample solution containing 400, 600 and 800 μg sample. This was done also for methanol (negative control) as a blank. These plates were kept for half an hour at a low temperature so that the test materials were diffused to the surrounding medium by this time. The plates were then incubated at 37 °C for 20 h. The diameter of the inhibition zone against each microorganism was measured by slide calipers and compared the results with standard antibiotic ciprofloxacin (5 μg/disc) and tetracycline (30 μg/disc) (positive control).

### Dilution method (MIC and MBC determination)

Minimum Inhibitory Concentrations (MIC) and Minimum Bactericidal Concentrations (MBC) assays were carried out on the essential oil by the macro-dilution method [21, 22]. The essential oil was dissolved in 30% dimethyl sulfoxide (DMSO) to obtain 10% (w/v) solution. For MIC test of the selected bacteria, the essential oil was first diluted in sterilized Mueller-Hinton broth in screw capped tubes containing broth medium in the concentration of 1000, 500, 250, 125, 62.5 and 31.25 μg/mL. Bacterial suspensions of the test organism were prepared in sterilized Mueller-Hinton broth. 1 mL of the dilution was added to each sterilized screw capped tube containing 1 mL of sample suitably diluted in the sterilized broth medium to give a final volume of 2 mL. A culture medium without sample (solvent DMSO) was used as a negative control. Ciprofloxacin (100–0.191 μg/mL.) was used as positive control. Tubes were incubated aerobically at 37 °C for 18 h and the growth was indicated by turbidity. The lowest concentration (preventing visible growth indicated by no turbidity) was identified as the MIC and expressed in μg/mL. The complete absence of growth was considered as the MBC [23]. To confirm the results of MBC, 10 μL of the experimental suspensions (withdrawn from the tubes with no growth) were subcultured on TSA (Tryptone Soy Agar) plates. The Plates were incubated at 37 °C for 24 h. It showed no bacterial growth, which was taken as the MBC. Values were recorded as μg/mL.

### Antifungal activity test

The antifungal activity test was performed by a similar procedure as antibacterial activity test. But PDA growth medium was used instead of NA medium. Positive control (Flucanozole 100 μg/mL) and essential oil containing petri dishs were incubated at 25 ± 2 °C for 72 h. The MICs and MBCs of the selected fungi were also done by the micro dilution method as described in the antibacterial activity test.

### Statistical analysis

Statistical analysis of all data was performed by means of the Microsoft Excel 7.0 version for windows. All data are presented as mean value ± standard deviation (*n* = 3). The values were considered significantly different at (*P* < 0.05).

## Results

### Proximate composition

The proximate composition of the *Anethum sowa* root L. is presented in Table 2. The root contained the high crude fibre, carbohydrate and ash with low fat and essential oil. The protein content is found to be moderate and also rich in glutamic acid, glycine and lysine. The moisture content was determined on the fresh weight basis, whereas the organic content was calculated on the dried basis. On the other hand, the water soluble ash was found the higher than the acid insoluble ash.

**Table 2:** Proximate composition of *Anethum sowa* L. Root

Characteristics	Percent (%) Composition
Moisture (On fresh weight basis)	78.46 ± 0.23
Dry matter (On fresh weight basis)	21.54 ± 0.23
Organic content	94.71 ± 0.12
Ash (On dry weight basis)	5.29 ± 0.10
Ash (On fresh weight basis)	1.14 ± 0.01
Acid insoluble ash	0.32 ± 0.02
Water soluble ash	1.87 ± 0.00
Nitrogen (Kjeldahl method)	0.32 ± 0.01
Protein	2.01 ± 0.07
Crude fibre	54.09 ± 0.50
Fatty oil (Hot extract)	1.14 ± 0.01
Fatty oil (Cold extract)	0.60 ± 0.11
Essential oil (On dry weight basis)	0.15 ± 0.09
Carbohydrate (Calculated by difference)	28.53 ± 0.30

Data are expressed as Mean ± SD (*n* = 3)

### Fatty acid composition

The cold and hot extracted fatty acids were semisolid, opaque, light golden colour with an unpleasant odour and a slightly bitter taste. The extracted fatty acids were found to be freely soluble in most of the common organic solvents. The fatty acid compositions are shown in Table 3. In all of them, 8 fatty acids were present. Amongst which, six fatty acids (1–3, 6–8) were found as saturated with a polyunsaturated and monounsaturated fatty acids in the cold and hot extracted part respectively. The most predominant fatty acids were palmitic acid followed by linoleic acid, behenic acid and lignoceric acid. Oleic acid was found the higher amount in hot extracted part than the cold extracted part. The nutritional quality of lipid fractions was evaluated by the different indexes (Table 3). In the present study, ratio of PUFA:SFA is 0.43:0.40 in the cold and hot extracted fatty acid respectively. In the current study, TI was found higher in cold extract than the hot extracted fatty acids. On the other hand, AI was found higher in the cold extract, but the lower value was found in hot extracted fatty acids. The standard and sample fatty acids methyl esters (FAMEs) chromatograms are shown in additional files (Additional file 1: Figure S1, Additional file 2: Figure S2 & Additional file 3: Figure S3).

**Table 3:** Fatty acid composition of *Anethum sowa* L. root extract (cold and hot extracts) by GC

Fatty acid compounds		Cold extract			Hot extract		
		R_*t*_	IA	Relative percentage	R_*t*_	IA	Relative percentage
1.	^a^Myristic acid (C_14:0_)	9.74	31956	5.24	9.751	407	0.17
2.	^a^Palmitic acid (C_16:0_)	13.33	205940	*33.81*	13.334	74176	*31.58*
3.	^a^Stearic acid (C_18:0_)	16.17	30015	4.93	16.176	9810	4.17
4.	^c^Oleic acid (C_18:1_)	15.83	5392	0.89	15.830	41976	*17.87*
5.	^b^Linoleic acid (C _18:2_)	15.75	182845	*30.03*	15.754	55875	*23.79*
6.	^a^Arachidic Acid (C_20:0_)	18.80	15831	2.60	18.809	4541	1.93
7.	^a^Behenic acid (C_22:0_)	21.24	77302	*12.70*	21.242	23580	*10.04*
8.	^a^Lignoceric acid (C_24:0_) (Tetracosanoic acid)	23.61	59715	*9.80*	23.612	24456	*10.45*
Total			608996	100		234821	100
^a^Saturated (%)				69.08			58.34
^b^Poly-unsaturated (%)				30.03			23.79
^c^Mono-unsaturated (%)				0.89			17.87
PUFA/SFA				0.43			0.40
AI				1.771			0.77
TI				2.84			1.72

*R*_*t*_ Retention time, *IA* Individual Area, *AI* Anthrogenic Index, *TI* Thrombogenicity Index, *PUFA* Poly-unsaturated fatty acids, *SFA* Saturated fatty acids

^a^Saturated, ^b^Poly-unsaturated, ^c^Mono-unsaturated

### Amino acid composition

The amino acid profile of *Anethum sowa* L. root was calculated in g/100 g protein on the dry weight basis and the results are shown in Table 4. In the present study, twelve amino acids were identified with an injection time of 35 min. Glutamic acid, lysine and glycine were found the most predominant amino acids in the analysed sample. The non-essential amino acid contained the higher amino acid content in comparison to the essential and neutral amino acids. Lysine was the highest essential amino acid out of seven essential amino acids. The total basic amino acid is greater than that of total acidic amino acid. The percentage of essential amino acids of the total amino acid was 45.90 whereas, total amount of sulphur containing amino acid was 5.49 g/100 g protein. On the other hand, only tyrosine was found as an aromatic amino acid in the current study. The standard and sample amino acids chromatograms are shown in additional files (Additional file 4: Figure S4 & Additional file 5: Figure S5),

**Table 4:** Amino acid composition (g/100 g crude protein) of *Anethum sowa* root

Name of Amino Acids	Sample Retention Time	Standard Retention Time	Sample Individual Area	Standard Individual Area	Concentration	
					g/100 g DB	g/100 g FB
Serine (Ser)^b,g^	10.392	10.471	1361824	2701518	3.58	4.01
Glutamic acid (Glu)^b,d^	*11.212*	*11.535*	*4493784*	*1649736*	*19.37*	21.67
Glycine (Gly)^b,g^	*15.262*	*16.138*	*14738830*	*7210517*	*14.53*	16.26
Alanine (Ala)^b,g^	17.360	18.191	525832	1186996	3.15	3.52
Valine (Val)^a,g^	21.543	19.752	356860	1517660	1.67	1.87
Methionine (Met)^a,f,g^	22.448	22.300	937009	1213589	5.49	6.14
Isoleucine (Ile)^a,g^	24.957	23.235	310181	1877608	1.17	1.31
Leucine (Leu)^a,g^	26.182	25.570	467152	1648575	2.01	2.25
Tyrosine (Tyr)^b,c,g^	27.415	26.796	390655	1877669	1.47	1.65
Histidine (His)^a,e^	28.371	28.295	7778337	8098543	6.82	7.64
Lysine (Lys)^a,e^	*32.084*	*32.045*	*3868261*	*1609813*	*17.08*	19.12
Arginine (Arg)^a,e^	33.663	33.693	473612	2266037	1.48	1.66
Total			35702337	32858261	77.87	87.14
^a^Essential amino acids					35.75	40.00
^b^Non Essen. Amino Acids					42.12	47.13
^c^Aromatic Amino Acids					1.47	1.65
^d^Acidic Amino Acids					19.37	21.67
^e^Basic Amino Acids					25.40	28.42
^f^Sulphur Amino Acids					5.49	6.14
^g^Neutral Amino Acids					33.10	37.04
% of essential amino acid in total amino acid					45.90	45.90

^a^Essential amino acids, ^b^Non essn. amino acids, ^c^Aromatic amino acid, ^d^Acidic amino acids, ^e^Basic amino acids, ^f^Sulphur amino acids, ^g^Neutral amino acids

### Elemental composition

The results of the elemental compositions of *Anethum sowa* L. root are shown in Table 5, Table 6 and Table 7. A total of 21 elements were analyzed which are designated as essential and non-essential elements. The relative standard deviation (RSD%) values for the studied elements were found to be ≤ 5%. The concentrations of the elements are calculated on fresh (FB) and dry weight basis (DB) within the significance level (p ≤ 0.05). In the current experiment, Ca, Mg, K, Na, Fe and Al were detected as rich essential elements followed by Ni, Se and Li on the dry weight basis (DWB). Hg was detected the highest level of the analyzed toxic elements followed by As and Cd. On the other hand, Cr and Pb were not found in the current experiment.

**Table 5:** Essential elemental composition (μg/kg) of *Anethum sowa* L. root

Elements	*Anethum sowa* L. Root			RSD %	Instrumental Method
	Solution Conc.	DWB (ppb)	FWB (ppb)		
Be	0.7977	2.09	1.90	2.84	ICP-MS
Ag	8.039	21.10	19.23	0.97	ICP-MS
Cs	8.2059	21.53	19.63	1.18	ICP-MS
Bi	8.2364	21.61	19.70	1.03	ICP-MS
Ga	9.6419	25.30	23.07	1.18	ICP-MS
Co	26.1657	68.67	62.61	1.36	ICP-MS
Li	121.093	317.84	289.76	0.08	ICP-MS
Se	348.141	913.79	833.06	2.11	ICP-MS
Ni	452.343	1187.30	1082.40	0.76	ICP-MS

*RSD* Relative Standard Deviation, *DWB* Dry Weight Basis, *FWB* Fresh Weight Basis

**Table 6:** Essential elemental composition (mg/kg) of *Anethum sowa* L. root

Elements	*Anethum sowa* L. Root			RSD %	Instrumental Method
	Solution Conc.	DWB (ppm)	FWB (ppm)		
Mn	6.56	17.21	15.69	0.1	AAS
Al	53.08	139.32	127.01	0.1	AAS
Fe	78.82	206.88	188.60	0.1	AAS
Na	110	288.72	263.21	0.1	FP
K	490	1286.15	1172.51	0.1	FP
Mg	2903.21	7620.33	6947.08	0.1	AAS
Ca	8991.18	23600.01	21514.95	0.1	AAS

*RSD* Relative Standard Deviation, *DWB* Dry Weight Basis, *FWB* Fresh Weight Basis

**Table 7:** Non-essential elemental composition (μg/kg) of *Anethum sowa* L. root

Elements	*Anethum sowa* L. Root			RSD %	Instrumental Method
	Solution Conc.	DWB (ppb)	FWB (ppb)		
Cr	ND	ND	ND	ND	ICP-MS
Pb	ND	ND	ND	ND	ICP-MS
Cd	14.7679	38.76	35.33	1.54	ICP-MS
As	16.5858	43.53	39.68	2.88	ICP-MS
Hg	48.45	965.52	880.22	0.3	AAS

*RSD* Relative Standard Deviation, *DWB* Dry Weight Basis, *FWB* Fresh Weight Basis

### Thermal analysis

The thermal analysis data are shown in Table 8. The thermal decomposition of the root powder comes through three common stages. In the first stage, broad and shallow endothermic DTA curve observed at 81.7 °C. In the second stage of decomposition is occurred on the DTA curve at 328.3 °C. A mass loss with a rate of 0.151 mg/min (at 75.9 °C) by heating the sample up to 0.373 mg/min (at 257 °C) is mentioned by the DTG curve. The highest mass loss was indicated in the second stage at 92.7% of TG at 275.7 °C and 1.167 mg/min at 328.3 °C of DTG curve. At this stage, pyrolysis was obtained when the temperature increased from 250 to 380 °C. Lignin decomposition was occurring in the range of 380–500 °C. The residual carbonaceous material in the previous steps was burnt at from 275 to 550 °C presenting an endothermic TG curve. The charred residue was produced by the destruction of low molecular compounds in the third and final stage of decomposition and yielded an ash residue. Finally, no mass loss was detected when the temperature increased from 550 °C to 800 °C. Representative thermo-gravimetric spectrum of the root is shown in additional file (Additional file 6: Figure S6).

**Table 8:** Results of TG and DTG (mass loss and temperature) of *Anethum sowa* L. root powder

Events	TG		DTG	
	Mass loss (%)	T_onset_-T_offset_ (^O^C)	Mass loss (mg/min)	T_onset_-T_offset_ (^O^C)
First step	92.7	T_room_-275	0.151	T_room_-75.9
Second step	53.3	276–327	0.373	76–257
Third step	29.6	328–358	1.167	258–328
% Residue	25.9	359–550	-	-

### Chemical composition of the essential oil

**Fig. 1:**
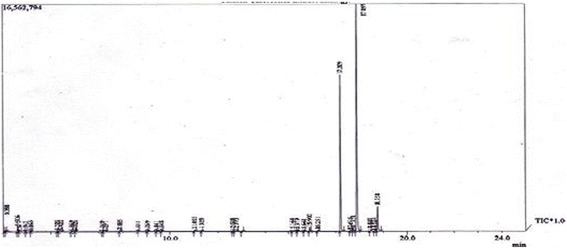
GC-MS Spectrum of *Anethum sowa* L. root essential oil

**Fig. 2:**
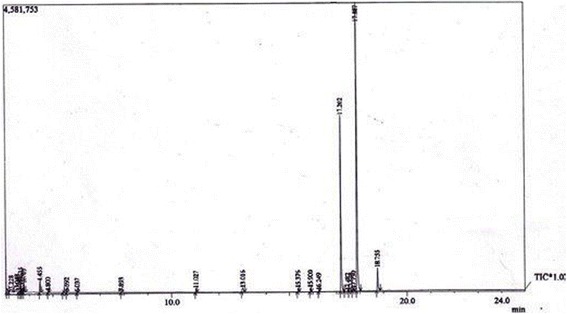
GC-MS spectrum of water extract part of *Anethum sowa* L. root

The essential oil of *A. sowa* was colourless with a characteristic odour. A total of 24 constituents were identified and quantified in the essential oil and its water extract part by GC and GC-MS and the results are shown in Tables 9 and 10 and Figs. 1 and 2. In this assay, 17 compounds from essential oil and 13 compounds from water extracted part were identified, which accounted for 100% of the total amount. Phenylpropanoids were contributed the major compounds of the essential oil and water extracted portion. Other predominant compound groups like lactone and ester were found in the water extracted part. Hydrocarbons were boiled out and produced an emulsion in the water extracted part which contributed 3.827% in the water extract part and 3.363% in the oily part. Apiol and m-diaminobenzene were the predominant components of the essential oil. In the case of water extracted part, apiol was found the highest component followed by m-diaminobenzene, β-butyrolactone and isobutylactone.

**Table 9:** Compound composition (% w/w) in the essential oil and water extract part of *Anethum sowa* L. root

SL No.	RI Index	Name of Compounds	Essential oil		Water extract part	
			R_*t*_	Conc. (%)	R_*t*_	Conc. (%)
1	349	Ethylene oxide	-	ND	16.249	0.294
2	653	Glycidol	-	ND	7.893	0.264
3	707	n-Pentanal	9.441	0.053	-	ND
4	721	Isobutyl acetate	-	ND	3.769	3.733
5	766	β-Butyrolactone	-	ND	3.228	5.131
6	767	2,3,3-Trimethyl hexane	3.677	0.068	-	ND
7	794	Toluene	3.606	2.40	3.635	3.327
8	806	n-Hexanal	4.165	0.166	-	ND
9	831	Furfural	-	ND	4.800	0.48
10	905	n-Heptanal	6.021	0.148	-	ND
11	907	p-Xylene	5.422	0.895	-	ND
12	1005	n-Octanal	7.885	0.238	-	ND
13	1081	2-Phenyl-acetaldehyde	8.653	0.106	-	ND
14	1197	p-Cymen-8-ol	11.026	0.878	11.027	1.109
15	1224	4-Thujen-2-alpha-yl acetate	11.323	0.082	-	ND
16.	1260	Cyclohexane, 3-hexyl	-	ND	17.730	0.50
17.	1294	2-Aminoundecane	15.164	0.244	-	ND
18.	1304	m-Diaminobenzene	18.758	10.446	18.755	8.778
19.	1334	(+/-)-Norephedrine	18.440	0.103	-	ND
20.	1421	Thymol acetate	12.773	0.878	-	ND
21	1516	Myristicin	15.900	0.868	15.900	0.716
22	1550	Elemicin	16.251	0.426	-	ND
23	1705	Apiol	17.897	*81.99*	17.887	*74.779*
24	2187	3,4-Dimethyl-benzoic acid 4-tert-bytyl-phenyl ester	-	ND	17.614	0.881
Total				100		100

*RI Index* Retention Index (compounds rearrangement with their retention index), *R*_*t*_ Retention time *ND* Not Detected

**Table 10:** Concentration of different compound classes of *Anethum sowa* L. root essential oil and water extractive part

Sl No.	Compound classes	Essential oil part (%)	Water extract part (%)
1.	Hydrocarbon	3.363	*3.827*
2.	Aldehyde	0.658	ND
3.	Alcohol	ND	0.264
4.	Lactone	ND	*5.131*
5.	Ester	0.96	*4.614*
6.	Oxide	ND	0.294
7.	Hemiterpenoid Aldehyde	0.053	0.48
8.	Monoterpenoid Alcohol	0.878	1.109
9.	Phenylpropanoid	*83.284*	*75.495*
10.	Others	*10.793*	*8.778*
Total		100	100

### Antioxidant activity on DPPH radical

**Fig. 3:**
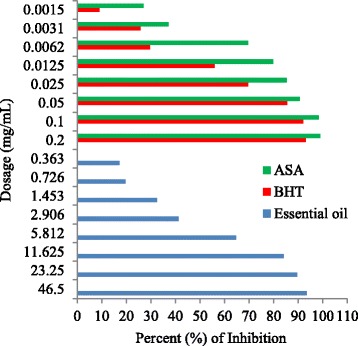
Scavenging activity of *Anethum sowa* L. root essential oil and standard in different dosage

The DPPH radical scavenging activity of the essential oil and synthetic standard ASA and BHT are shown in Table 11 and Fig. 3. The IC_50_ value of the essential oil was found to be 6.13 ± 0.03 mg/mL with the highest inhibition of 93.43 mg/mL. However, this value is much weaker than those of standard BHT and ASA.

**Table 11:** Antioxidant activity of *Anethum sowa* L. root essential oil and standards

Essential oil			Standard Antioxidants			
Dose Concentration (mg/mL)	Log Dose	Average percent of Inhibition ± SD	Dose Concentration (μg/mL)	Log Dose	Average percent of Inhibition ± SD of BHT	Average percent of Inhibition ± SD of ASA
46.5	1.66	93.43 ± 0.24	200	2.301	93.02 ± 0.50	99.05 ± 0.21
23.25	1.36	89.56 ± 0.22	100	2	91.98 ± 0.19	98.35 ± 0.17
11.625	1.06	84.17 ± 0.25	50	1.698	85.51 ± 0.76	90.71 ± 0.60
5.8125	0.764	64.68 ± 0.21	25	1.397	69.54 ± 0.60	85.31 ± 0.60
2.90625	0.463	41.25 ± 0.12	12.5	1.096	55.87 ± 0.82	79.79 ± 0.18
1.453125	0.162	32.41 ± 0.31	6.25	0.795	29.69 ± 0.97	69.67 ± 0.68
0.72656	−0.138	19.71 ± 0.40	3.125	0.494	25.63 ± 0.51	37.26 ± 1.12
0.36328	−0.439	17.18 ± 0.05	1.5625	0.193	9.05 ± 0.51	27.02 ± 1.19
Average IC_50_ value = 3.07 ± 0.01 mg/mL					IC_50_ value = 11.84 ± 0.29 μg/mL	IC_50_ value = 3.74 ± 0.05 μg/mL

*ASA* Ascorbic acid, *BHT* Tert-butyl-1-hydroxytoluene, Mean ± SD

### Brine shrimp cytotoxicity assay

**Fig. 4:**
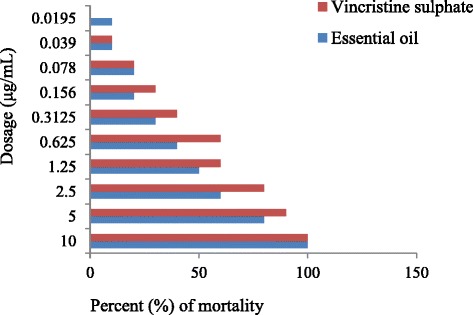
Cytotoxic activity of *Anethum sowa* L. root essential oil and vincristine sulphate against different dosage

The results of brine shrimp cytotoxicity lethality test of the essential oil were tabulated by the Finney probit method [24] and shown in Table 12 and Fig. 4. The essential oil was found to be toxic by comparing with standard anticancer drug vincristine sulphate.

**Table 12:** Effect of vincristine sulphate and essential oil of *Anethum sowa* L root on brine shrimp cytotoxicity assay

Dose (μg/mL)	Log Dose (μg/mL)	% of mortality		Probit		LC_50_ (μg/mL)	
		Vincristine sulphate	Root essential oil	Vincristine sulphate	Root essential oil	Vincristine sulphate	Root essential oil
10	1	100	100	7.0959	6.723		
5	0.699	90	80	6.2802	5.8408		
2.5	0.3979	80	60	5.8413	5.241		
1.25	0.0969	60	50	5.2402	*4.9977*		
0.625	−0.2041	60	40	*4.9992*	4.7482	*0.48*	*0.81*
0.3125	−0.5051	40	30	4.7467	4.4782		
0.156	−0.8069	30	20	4.4774	4.1638		
0.078	−1.1079	20	20	4.1665	4.1753		
0.039	−1.4089	10	10	3.7206	3.7198		
0.0195	−1.71	0	10	0	3.8288

### Antimicrobial activity of *Anethum sowa* L. root essential oil

**Fig. 5:**
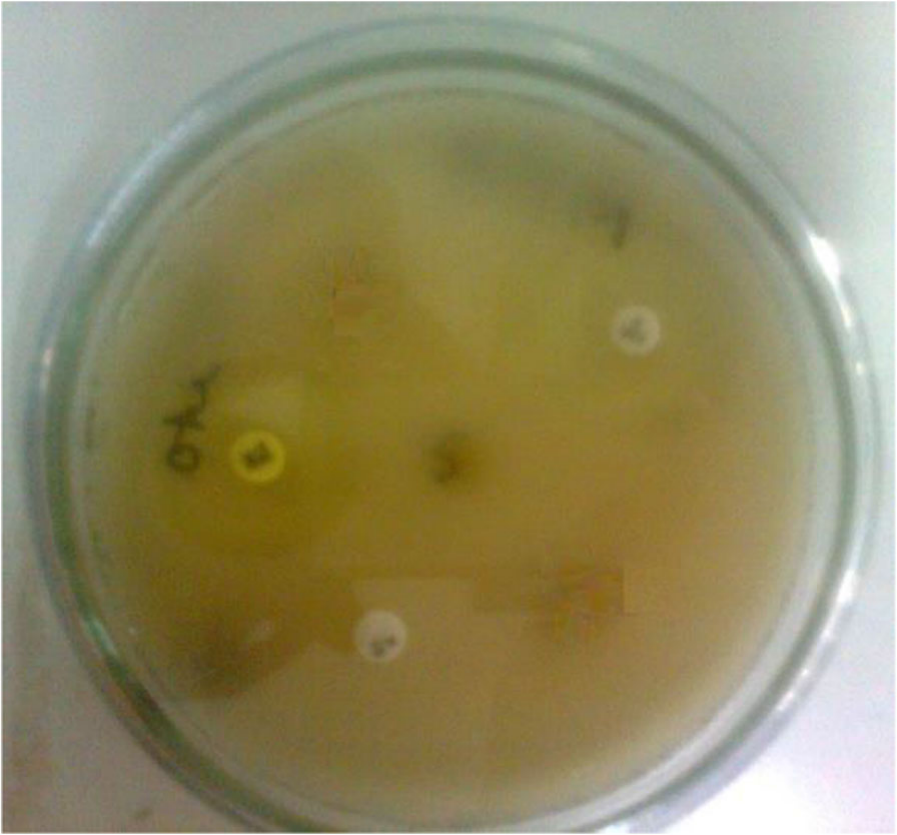
Zone of *Bacillus cereus* for standard, and essential oil at 800 μg/ well

**Fig. 6:**
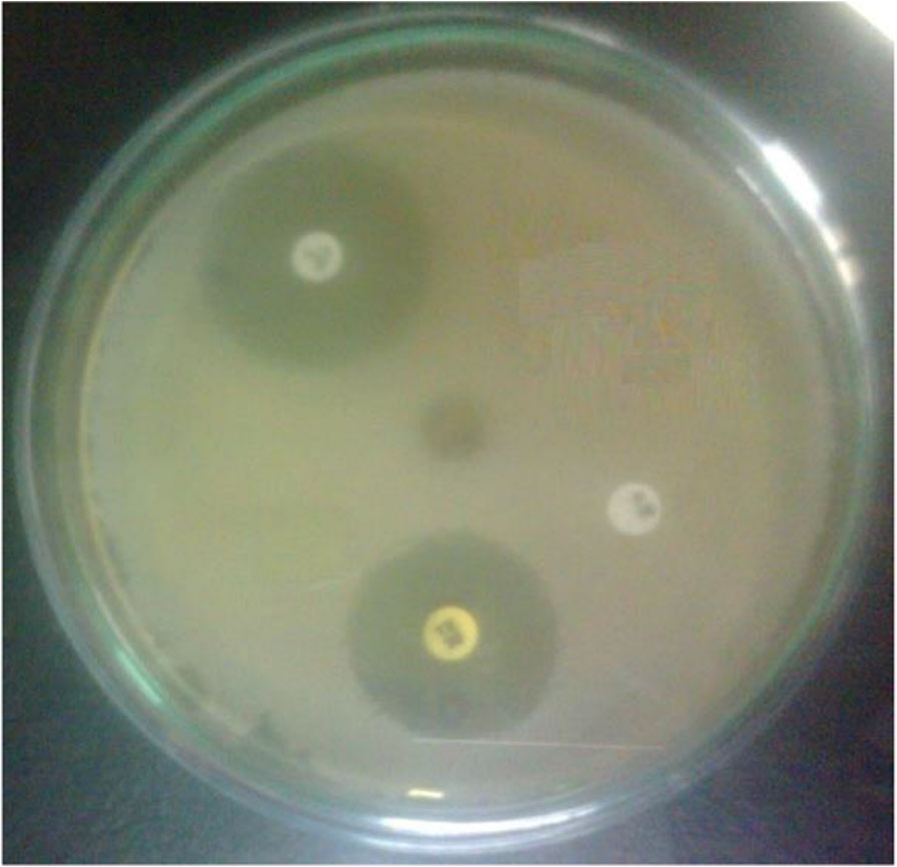
Zone of *P. aeruginosa* for standard and essential oil at 800 μg/ well

The results of antimicrobial activity of *Anethum sowa* L. essential oils are shown in Tables 13, 14 and Figs. 5 & 6. The oil inhibited the growth of bacterial strains producing a zone diameter of inhibition from 3 to 16 mm, depending on the susceptibility of the tested bacteria and the different dosage level. The highest inhibition of zone was found against *Escherichia coli 2799* followed by *Pseudomonas aeruginosa* and their effects are also higher than standard antibiotic tetracycline and lower than ciprofloxacin. On the other hand, *Enterococcus faecalis* showed the high resistant to the essential oil. However, other Gram-positive and Gram-negative bacteria exhibited moderate to weak activities than the standard antibiotics. The MICs of the essential oils were within concentration ranges from 62.5 to 250 μg/mL and the respective MBCs were from 125 to 500 μg/mL. The organism like *E. coli* 12079 and *S. typhi* were less susceptible to the oil with MICs value of 250 μg/mL respectively. The MIC and MBC values of standard ciprofloxacin were varied between 0.19–6.25 μg/mL and 3.125–100 μg/mL respectively.

**Table 13:** Anti-bacterial activity (Zone of Inhibition) of *Anethum sowa* L. root essential oil by diffusion method

Test organisms		Zone of Inhibition in mm				
		400 μg/well	600 μg/well	800 μg/well	CP	TE
Gram-positive bacteria	*Bacillus subtilis*	nz	4	7	27	24
	*Bacillus cereus*	5	7	8	29	21
	*Staphylococcus aureus*	nz	3	7	25	26
	*Enterococcus faecalis*	nz	nz	nz	20	16
Gram -negative bacteria	*Escherichia coli 12079*	nz	4	7	25	9
	*Escherichia coli 2799*	11	13	16	23	11
	*Pseudomonas aeruginosa*	8	9	11	29	16
	*Salmonella enteritidis* 1375	nz	nz	5	39	23
	*Salmonella typhi*	nz	5	9	35	25
	*Acetobacter aceti*	nz	nz	3	28	23

*CP* Standard Ciprofloxacin (5 μg/disc), *TE* Standard Tetracycline (30 μg/disc)

**Table 14:** Minimum inhibitory concentration (MIC) and Minimum bactericidal concentration (MBC) of *Anethum sowa* L. root essential oil and standard ciprofloxacin

Test organisms		Essential Oil		Std. CP	
		MIC (μg/mL)	MBC (μg/mL)	MIC (μg/mL)	MBC (μg/mL)
Gram-positive bacteria	*Bacillus subtilis*	125	250	1.55	12.5
	*Bacillus cereus*	125	500	3.125	25
	*Staphylococcus aureus*	125	250	6.25	50
	*Enterococcus faecalis*	62.5	125	0.37	100
Gram -negative bacteria	*Escherichia coli 12079*	250	500	0.75	50
	*Escherichia coli 2799*	62.5	125	0.37	6.25
	*Pseudomonas aeruginosa*,	62.5	125	3.125	25
	*Salmonella enteritidis* 1375	62.5	250	0.37	6.25
	*Salmonella typhi*	250	500	0.19	3.125
	*Acetobacter aceti*	62.5	500	0.37	50

*Std. CP* Standard Ciprofloxacin

### Antifungal activity of *Anethum sowa* L. root essential oil

The antifungal activity of the essential oil shown in Table 15. The highest zone of inhibition was shown against *A. niger* at 800 μg/well but there was no observable zone of inhibition was found in *T. sp*. On the other hand, no zone was observed at 400 μg/well dose level. The standard drug fluconazole was highly sensitive to all the fungi at the dose of 100 μg/well. In the MICs and MBCs experiments, the essential oil showed the highest inhibition i.e. the lowest MICs and MBCs. In the case of fluconazole, it completely inhibited the entire test fungi.

**Table 15:** Anti-fungal activity of *Anethum sowa* L. root essential oil

Fungi	Zone of Inhibition in mm					
	Essential oil			Fluconazole	MIC/MBC (μg/mL)	
	400 μg/well	600 μg/well	800 μg/ well	100 μg/well	Essential oil	Fluconazole
*Candida albicans*	-	3	7	23	125/500	62.5/250
*Aspergillus niger*	-	5	9	16	250/500	125/500
*Sacharomyces cerevaceae*	-	4	7	10	62.5/250	62.5/250
*Trichoderma sp.*	-	-	-	9	250/500	125/500

## Discussion

The data on the proximate composition of *Anethum sowa* L. root has been reported for the first time. The ash content is found very high in the current study, indicating the high quality of essential minerals which is comparable with an acceptable ash range of legumes (2.4–5.0%) [25]. Acid insoluble ash is an indicator of silicate impurity and water soluble ash is indicated the highly soluble mineral contents in the root sample.

The oil from a single source has not been found to be suitable for consumption and medicinal preparation because of their different chemical compositions. So, the necessity for searching of new oil source is a major research interest in the present day. Our previously published data on the fatty acid composition of *Anethum sowa* seed and flower part [26, 27] revealed that oleic acid was the highest in seed (87.10%) and flower (51.93%) part than our current study. Moreover, palmitic acid content is almost similar to the flower fatty acid (30.74%) but it is found higher than the seed (4.27%) fatty acid. Linoleic acid is one of the essential polyunsaturated fatty acids which prevents cardiovascular diseases and its derivatives serve as structural components of the plasma membrane [28]. It also has a beneficial effect on blood lipids, lowering blood pressure and serum cholesterol. The nutritional value of linoleic acid is due to its metabolism at the tissue levels, which produced the hormone-like prostaglandins [29]. It was found the second highest fatty acid in the present study. The saturated fatty acids like lauric, myristic and palmitic acid have been established as the most important of the dietary risk factors and resulted in a high level of total blood cholesterol [30]. Diets with a PUFA:SFA ratio was found the below of 0.45, which considered inadequate because of their potential to increase blood cholesterol levels [31]. On the other hand, higher SFA stimulates the *de nova* synthesis of cholesterol in the body. Thus, it indicates that these are not a good source of fatty acids for human health. Moreover, TI was found higher than 1 in cold and hot extracted fatty acids while AI was found higher than 1 in cold extracted fatty acid, but the lower value was found in hot extracted fatty acids. The AI and TI indexes have considered as cardiovascular disease risk factors. Thus, these indices must be kept low. It was reported that lower AI leads to a decrease in the total cholesterol and the LDL-cholesterol in human blood plasma [32]. To the best our knowledge, this is the first report regarding the fatty acids of *A. sowa* root. This investigation has given us valuable information about the structure of the fatty acids and also indicated that these oils cannot be considered as functional ingredients of human diet but it may be a valuable source for the cosmetic or industrial application.

Primarily, the nutritive value of a protein depends on the capacity to satisfy the needs for nitrogen and essential amino acid. It is indicated that the *A. sowa* L. root protein is probably basic in nature. There is a dearth of information regarding the amino acid profile of the *Anethum sowa* L. root sample to the best our knowledge. Lisiewska (2004) [33] reported that leaves of *Anethum graveolens* L. contained rich in essential amino acid than the whole plant where leucine (10.10 for the whole plant and 9.62 for leaves of g/100 g protein) was the highest essential amino acid. Some amino acids like tryptophan, asparagines and glutamine were destroyed by acid hydrolysis method and therefore these acids were not determined. Cystein was not determined because of the rapid oxidation of this compound to form cysteic acid. The total sulphur containing amino acid (5.49 g/100 g protein) was close to having the recommended protein value (5.8 g/100 g) for infants [34]. This value was well above the 39%, considered to be adequate for ideal protein for infant, 26 g/100 g protein for children and 11 g/100 g protein for adult [34]. The storage of protein (i.e. amino acids) in the different parts of the plant is readily used for germination and seedling growth [35]. The proteins are considered for its essential amino acid profile due to the inability of the human body to synthesize them. The amino acid profile of the studied root sample suggests that its protein is beneficial for human health.

The concentration of elements is not uniformly distributed throughout the plant. In general, the roots contain the highest level of elements followed by stems, leaves, flowers, buds and fruits of the different maturity stages. Nevertheless, uptake of elements by a plant is influenced by various factors, including the type of plant, nature of the soil, climate and agricultural practices [36]. In the current study, the different elemental concentrations were varied due to the above factors. *A. sowa* L. root accumulated a significant amount of minerals in Bangladeshi geo-environmental condition. The results are also compared with the reported [8] data of *Anethum graveolens* L. root where K (38,500 ppm) had the highest value, Ca (12,200 ppm) and Mg (6700 ppm) had the lowest values than our current study. The elements, especially Ca, Mg, K, Na, Fe and Al play a significant role in human metabolism and the life processes. The rich amount of Ca is important because of its role in bones, teeth, muscle system and heart functions [37]. Mg has been particularly shown to play a significant role as a regulatory cation in direct and indirect traumatic brain injury [38]. Another report on Mg, it also helps in preventing some heart disorders and high blood pressure [39]. Recently, it has been reported that trace amounts of Rb and Cs help in the breakdown of starch to glucose [36]. Cs was present in the trace amount (21.53 ppb) in the present study. Se has anti-oxidising function and it is essential for providing the organism with triiodothyroxine produced from thyroxine [40]. It contributes to the maintenance of cellular antioxidative balance when taken up at the recommended dietary allowance of 50–100 μg/day and tolerable upper intake limit is 400 μg/day [41]. The high dosage of selenate has been shown to normalize hyperglycemia [36]. Li is another element with beneficial pharmacological properties which effectively used in the treatment of manic depressive disorders [42]. The result also showed that many of these elements have vital importance in human metabolism for growth, developments and also prevention and healing of diseases. The non-essential or toxic elemental contents in spice and medicinal plants are attracting attention to the scientist and also the food chemist due to its adverse effect on the human body in respect of medicinal and food preparation. The permissible limits of Hg and As are 1 and 10 mg/kg in foodstuff respectively [43]. The maximum prescribed limit of Cd is 0.3 mg/kg for herbal medicines and its product while the dietary intake limit is 10.3 mg/kg as per who recommendation [44]. Moreover, the toxic elements were found below the prescribed limits in the present experiment. Nonetheless, excessive intake can cause poisoning in the human body. Hg poisoning can result in damage to the brain, kidney, lungs including acrodynia (pink disease), Hunter-Russell syndrome and Minamata disease [45]. The acute sign of As poisoning includes fever, anorexia, hepatomegaly, cardiac arrhythmia, transient encephalopathy and irritation of the gastrointestinal tract [46]. Cd intoxication can lead to kidney, bone and pulmonary damages [47]. Pb and Cd cause both acute and chronic poisoning, adverse effects on the kidney, liver, heart, vascular and immune system [48]. Furthermore, root parts of *Anethum sowa* L. herb is not usually consumed, it is wasted mostly. The present investigation will be helpful for Ayurvedic and pharmaceutical industries to assess the essential and toxic elemental level for medicinal preparation.

Thermo-gravimetry is a technique in which change in the weight of a substance is recorded as a function of temperature or time. This technique is useful for the pre-formulation study of herbal preparation through physical and chemical changes. It detects the impurity, identified the complex mixture and thermal stability of the plant samples [49]. In the first stage, broad and shallow endothermic effect on the DTA curve indicated a small loss in mass by a dehydration reaction. This peak is due to desorption of moisture of the root powder material together with the evaporation of volatile components or essential oils. The chemical reaction was occurring in the second stage of decomposition by a strong exothermic effect on the DTA curve. A mass loss of the DTG curve was verified due to the elimination of moisture retained in this material. In the second stage, the highest mass loss was reflected by the TG and DTG curves which are due to the destruction and combustion of compounds contained in the root samples. The behaviour of the pyrolysis curve indicated hemi-cellulose and cellulose decomposition, as well as the loss of remaining adsorbed moisture. Lignin decomposition indicated that this structure presents a higher stability than hemi-cellulose and cellulose. The last stage indicated that the presence of oxides (mainly those of aluminium and silicon), which were stable at higher temperatures. The last stage was also associated with a large exothermic effect.

The essential oils and extracts of the medicinal plants have been used for food preservation, scenting agent and as a traditional healthcare since thousands of years [50]. In the GC-MS analyses, apiol and m-diaminobenzene were found the major constituents of *A. sowa* root essential oil. It was reported that apiol (20%) was found as the main compound in the root essential oil of *Anethum graveolens* L. [7]. In the earlier studies, phenylpropanoid compounds were the rich level in leaf and stem part of *A. sowa* L. [51, 52]. On the other hand, apiol was also found in some umbelliferae plants like parsley (*Petroselinum sativum* and *Petroselinum crispum*) which have accounted for 18.23 and 17.54% respectively [53, 54]. Moreover, m-diaminobenzene (68.2%) was also found as the highest amount in other *Apiaceae* plant (*Ligusticum jeholense*) essential oil [55]. On the other hand, carvone (27.8%) was found the main predominant compound in *Anethum graveolens* L. followed by limonene (15.5%), α-phellandrene (14.7%) and apiol (3.1%) [9] while carvon, limonene and α-phellandrene were totally absent in the present study. The variations of oil composition with *Anethum graveolens* species may be due to plant genetic base, development and mostly different environmental conditions.

The oil exhibited marked DPPH free radical scavenging activity in a concentration-dependent manner. The antioxidant activity of the oil was evaluated by the decolorisation of stable DPPH radical due to its hydrogen donating ability. The absorption is stoichiometric with respect to the number of electrons taken up [56].

Apiol is the major component of the essential oil and it has contained two methoxy groups in the symmetrical position of the benzene ring. This methoxy (−OCH_3_) group is a well-known strong electron donating group, which can increase the stability of the benzene ring and enhance the radical scavenging activity as a result [54]. Zhang [54] and Elisia [57] indicated that the antioxidant activity of apiol is much stronger than the myristicin. It was also reported that substitution on the indole benzene ring with a methoxy group had greatly enhanced the radical scavenging activity of the un-substituted compounds [58]. The addition of a methoxy group also confers antioxidant activity to the chalcones [59]. Therefore, the essential oil and its king compound apiol have contributed significantly the antioxidant property being reported for the first time. On the basis of present findings, the essential oil of *Anethum sowa* L. root may be the potent alternative as natural antioxidants.

The brine shrimp cytotoxicity of the plant extract represents a rapid, inexpensive and simple bioassay technique for cytotoxic and anti-tumor activity. This test is also considered to be phototoxic, pesticidal, trypanocidal, antitumor, anticancer, enzyme inhibition and ion regulation activities. The assay indicated that essential oil is half of the vincristine sulphate toxicity. The apiol was reported to decrease the proliferation of human colorectal carcinoma cells (COLO 205) [60]. Moreover, the acaricidal constituent of apiol was isolated by various chromatographic techniques from *Petroselinum sativum* seeds and showed very strong acaricidal activity. This activity could be related to allyl (−C_3_H_5_) and methoxy (−OCH_3_) functional groups [61]. The antiproliferative activity of apiol and myristicin of *Athamanta sicula* L. also reported on human chronic myelogenous leukemia, lung tumor and breast adenocarcinoma cell line activities [62]. Nonetheless, apiol is known to have powerful insecticidal and synergist effect [6]. Therefore, the cytotoxic activity of the essential oil is due to the apiol compound of the *A. sowa* root. Furthermore, this essential oil may be helpful for treatment of premature ageing and cancer therapy by preventing oxidative damage through lipid peroxidation. Though it is the first report of cytotoxic activity on the brine shrimp, extensive studies are to be needed to assess the anticancer activity on the cell line of the essential oil and its isolated compound apiol.

The antimicrobial activities are responsible for oxygenated monoterpenes [63]. The appreciable antimicrobial activity of the essential oil of *Anethum sowa* L. may be attributed to the presence of monoterpenes compounds in the oil. On the other hand, the activity of the essential oil can be explained by the chemical structure of the major constituents of the oil. Apiol has a nucleus containing a polar functional group that is known to form hydrogen bonds with the active sites of the target enzyme [64]. It is assumed that the antimicrobial activity may be responsible for its apiol constituents, on the basis of that mechanism. The Gram-positive bacteria are generally more sensitive to the spice and herb essential oil than Gram-negative bacteria due to an outer membrane and a unique periplasmic space in the Gram-positive bacteria [65]. The essential oil degrades the cell wall of the organism by interacting with the essential oil component which causes disrupt cytoplasmic membrane and damage membrane protein [66]. However, it did not completely follow the trend described above and showed the potent activity against Gram-negative bacteria. Nonetheless, the antimicrobial activity of essential oils may be due to the synergistic effect of the major compounds of the oil and also combined with another minor constituent. The results of the standard antibiotic showed the stronger activity against most of the bacterial species than the essential oil.

In the present study, the essential oils inhibited the fungal growth but their effectiveness varied. On the other hand, the essential oil was found to be more effective against fungi of the MIC and MBC assays. This activity may be responsible for the high content of apiol along with other minor constituents in the oil. Further investigations are needed to be carried out for the better understanding of the present issue.

## Conclusion

As the chemical composition, as well as the pharmacological study of *Anethum sowa* L. root, have not been reported previously, it was undertaken for the present investigation. In the present results, we have found the *Anethum sowa* L. root as a rich source of mineral constituents along with amino acids. Inorganic elements remain complexed with organic ligands and make them bioavailable to the body system. The essential amino acids of the protein may prove its potentiality in medicinal preparation. The thermal analysis suggested that it is a simple, effective and rapid method to characterize the *Anethum sowa* L. species as well as to evaluation of phytotherapy. The current study of the essential oil showed weak antioxidant activity compared to ASA and BHT and also exhibited moderate antibacterial activity against Gram-negative bacteria than Gram-positive bacteria and fungi. This study contributes to knowledge of the chemical properties and also the biological significance of essential oil of *Anethum sowa* L. root as an aromatic, spice and medicinal plants. Several compounds of different polarity present in small amounts can also contribute to the biological activity. The results of the present studies will be helpful for herbal formulation and also for the basis of further studies.

## Additional files

Additional file 1: Figure S1. GLC spectrum of standard fatty acids methyl esters (FAMEs). (DOCX 132 kb)
Additional file 2: Figure S2. GLC spectrum of pet.ether extract of *Anethum sowa* L. root. (DOCX 139 kb)
Additional file 3: Figure S3. GLC spectrum of hexane extract of *Anethum sowa* L. root. (DOCX 149 kb)
Additional file 4: Figure S4. Chromatogram of standard amino acids. (DOCX 108 kb)
Additional file 5: Figure S5. Amino acids chromatogram of *Anethum sowa* L. root. (DOCX 201 kb)
Additional file 6: Figure S6. Thermo-gravimetric spectrum of *Anethum sowa* L. root powder. (DOCX 125 kb)

## Abbreviations

AAS: Atomic Absorption Spectrometry
AI: Atherogenic Index
AOAC: Association of Official Analytical Chemist
BHT: Tert-butyl-1-hydroxytoluene
DMSO: Dimethyl Sufoxide
DTA: Differential Thermal Analysis
DTG: Derivative Thermo Gravimetric
DWD: Dry Weight Basis
FAMEs: Fatty Acid Methyl Esters
GC-MS: Gas Chromatography and Mass Spectroscopy
ICP-MS: Inductively Coupled Plasma Mass Spectrometry
MBC: Minimum Bactericidal Concentration
MIC: Minimum Inhibitory Concentration
NA: Nutrient Agar, ASA: Ascorbic acid
PDA: Potato Dextrose Agar
PUFA: Poly Unsaturated Fatty Acid
R_*t*_: Retention time
SFA: Saturate Fatty Acids
TGA: Thermo gravimetric analysis
TI: Thrombogenic Index
