# Supplementary figures and images for: Chemical composition and pharmacological significance of Anethum Sowa L. Root
Source: BMC Complement Altern Med. 2017 Feb 23;17:127. doi: 10.1186/s12906-017-1601-y (PMC5324201; doi:10.1186/s12906-017-1601-y)

**Addtional file**

**
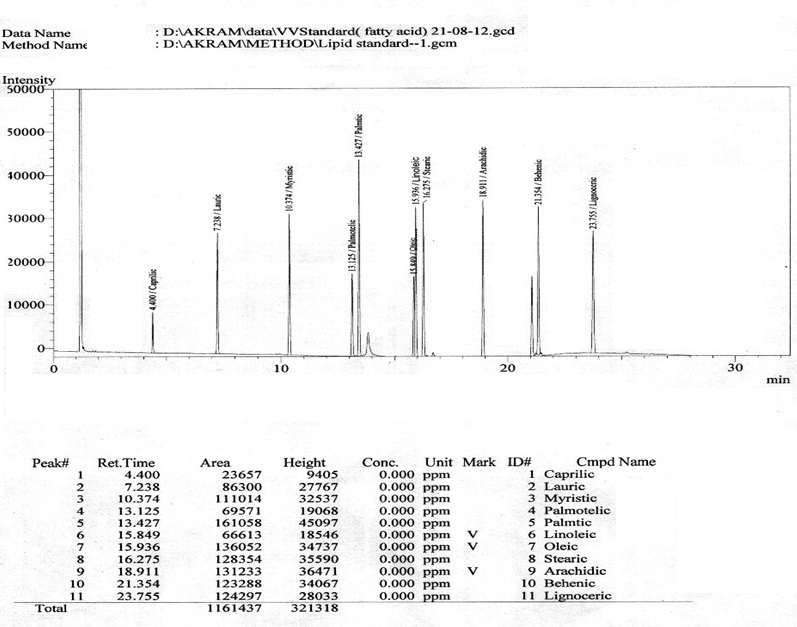
**

**Figure S1** GLC spectrum of standard fatty acids methyl esters (FAMEs)

Supplement: Additional file 1: Figure S1. — GLC spectrum of standard fatty acids methyl esters (FAMEs). (DOCX 132 kb) [file 12906_2017_1601_MOESM1_ESM.docx]

**Additional file**

**
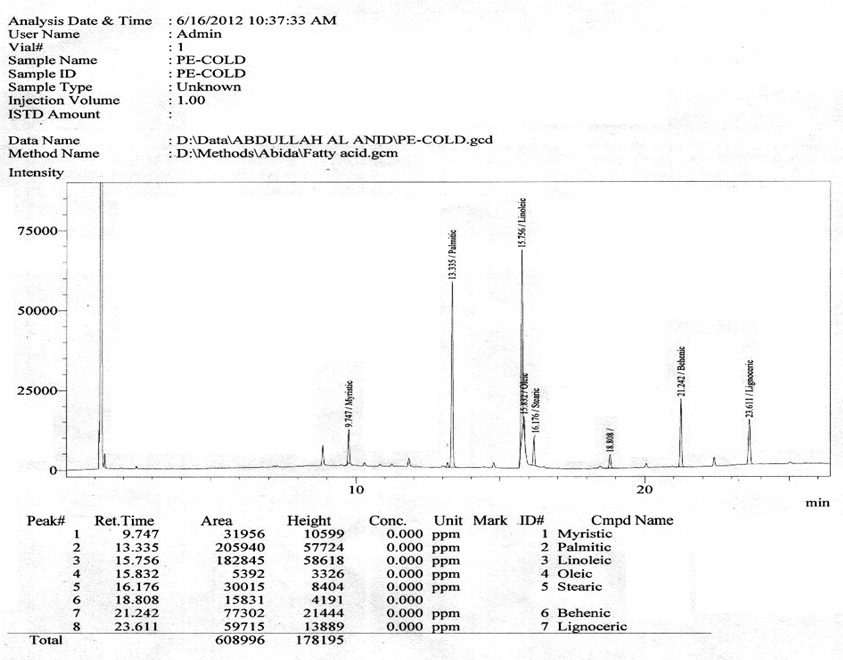
**

**Figure S2** GLC spectrum of pet.ether extractof *Anethum sowa* L. root.

Supplement: Additional file 2: Figure S2. — GLC spectrum of pet.ether extract of Anethum sowa L. root. (DOCX 139 kb) [file 12906_2017_1601_MOESM2_ESM.docx]

**Additional file**

**
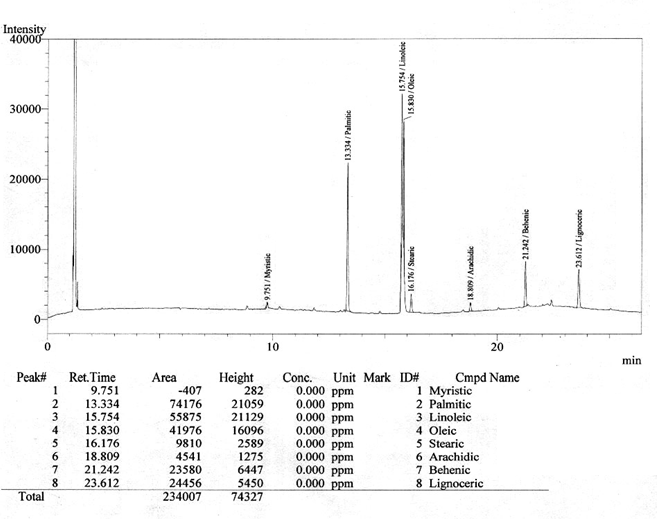
**

**Figure S3** GLC spectrum of hexane extractof *Anethum sowa* L. root

Supplement: Additional file 3: Figure S3. — GLC spectrum of hexane extract of Anethum sowa L. root. (DOCX 149 kb) [file 12906_2017_1601_MOESM3_ESM.docx]

**Additional file**


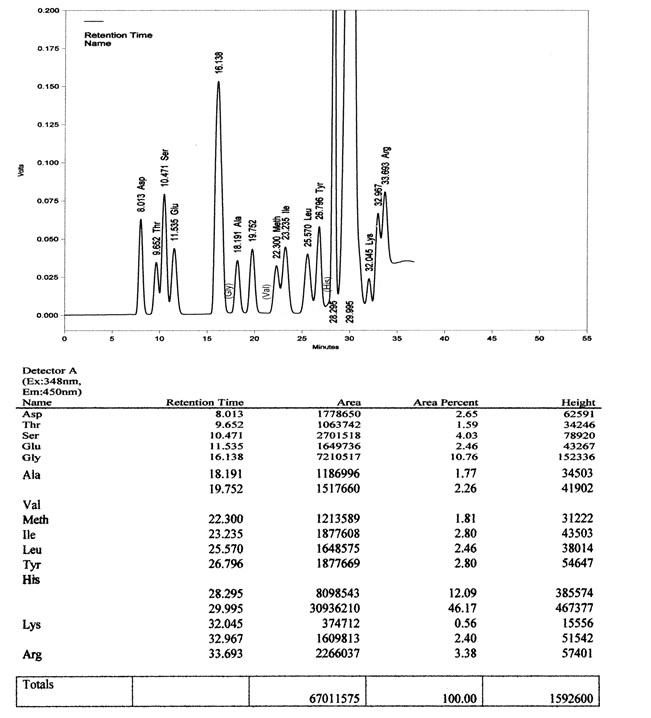


**Figure S4** Chromatogram of standard amino acids.

Supplement: Additional file 4: Figure S4. — Chromatogram of standard amino acids. (DOCX 108 kb) [file 12906_2017_1601_MOESM4_ESM.docx]

**Additional file**


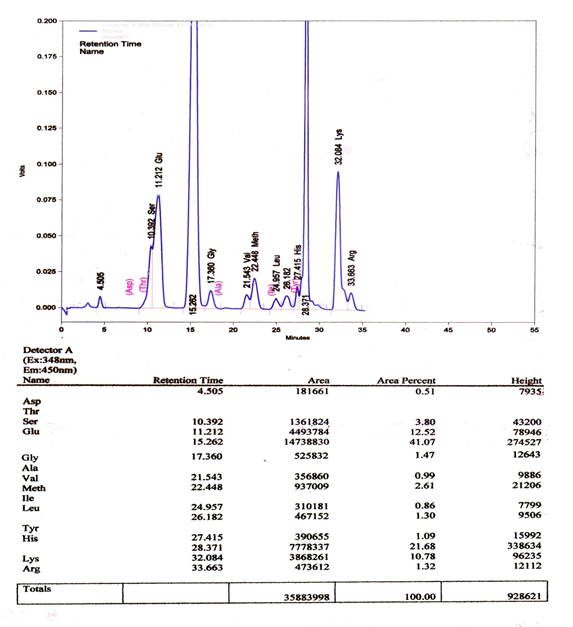


**Figure S5** Amino acids chromatogram of *Anethum sowa* L. root.

Supplement: Additional file 5: Figure S5. — Amino acids chromatogram of Anethum sowa L. root. (DOCX 201 kb) [file 12906_2017_1601_MOESM5_ESM.docx]

**Additional file**

**
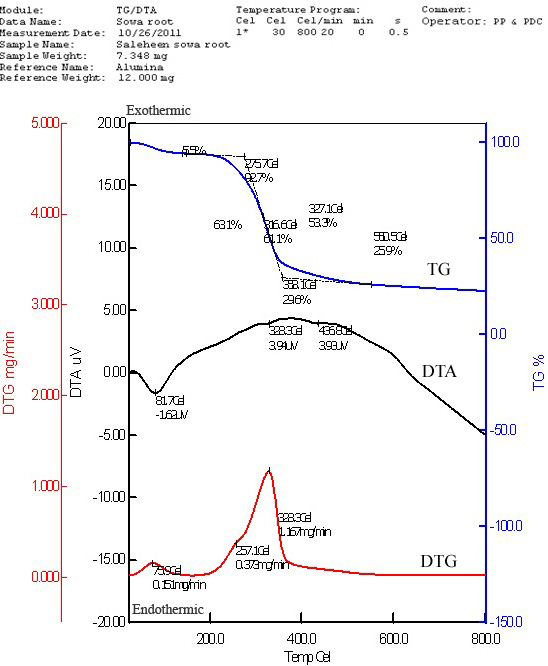
**

**Figure S6** Thermo-gravimetric spectrum of *Anethum sowa* L. root powder.

Supplement: Additional file 6: Figure S6. — Thermo-gravimetric spectrum of Anethum sowa L. root powder. (DOCX 125 kb) [file 12906_2017_1601_MOESM6_ESM.docx]
